# *Cayratia albifolia* C.L.Li exerts anti-rheumatoid arthritis effect by inhibiting macrophage activation and neutrophil extracellular traps (NETs)

**DOI:** 10.1186/s13020-024-00910-4

**Published:** 2024-03-05

**Authors:** Wei Wang, Zai-Qi Zhang, Yi-Chi Zhang, Yi-Qiang Wu, Zhuo Yang, Yong-Zhe Zheng, Jia-Hong Lu, Peng-Fei Tu, Ke-Wu Zeng

**Affiliations:** 1 State Key Laboratory of Natural and Biomimetic Drugs, School of Pharmaceutical Sciences, Peking University, Beijing, 100191 China; 2 Hunan Provincial Key Laboratory of Dong Medicine, Hunan University of Medicine, Huaihua, 41800 China; 3 State Key Laboratory of Quality Research in Chinese Medicine, Institute of Chinese Medical Sciences, University of Macau, Macau, 999078 SAR China

**Keywords:** Rheumatoid arthritis, *Cayratia albifolia* C.L.Li, Macrophage, PI3K-Akt pathway, NETosis

## Abstract

**Background:**

*Cayratia albifolia* C.L.Li (CAC), commonly known as “Jiao-Mei-Gu” in China, has been extensively utilized by the Dong minority for several millennia to effectively alleviate symptoms associated with autoimmune diseases. CAC extract is believed to possess significant anti-inflammatory properties within the context of Dong medicine. However, an in-depth understanding of the specific pharmaceutical effects and underlying mechanisms through which CAC extract acts against rheumatoid arthritis (RA) has yet to be established.

**Methods:**

Twenty-four Sprague–Dawley rats were divided into four groups, with six rats in each group. To induce the collagen-induced arthritis (CIA) model, the rats underwent a process of double immunization with collagen and adjuvant. CAC extract (100 mg/kg) was orally administered to rats. The anti-RA effects were evaluated in CIA rats by arthritis score, hind paw volume and histopathology analysis. Pull-down assay was conducted to identify the potential targets of CAC extract from RAW264.7 macrophage lysates. Moreover, mechanism studies of CAC extract were performed by immunofluorescence assays, real-time PCR and Western blot.

**Results:**

CAC extract was found to obviously down-regulate hind paw volume of CIA rats, with diminished inflammation response and damage. 177 targets were identified from CAC extract by MS-based pull-down assay. Bioinformatics analysis found that these targets were mainly enriched in macrophage activation and neutrophils extracellular traps (NETs). Additionally, we reported that CAC extract owned significant anti-inflammatory activity by regulating PI3K-Akt-mTOR signal pathway, and inhibited NETosis in response to PMA.

**Conclusions:**

We clarified that CAC extract significantly attenuated RA by inactivating macrophage and reducing NETosis via a multi-targets regulation.

**Graphical Abstract:**

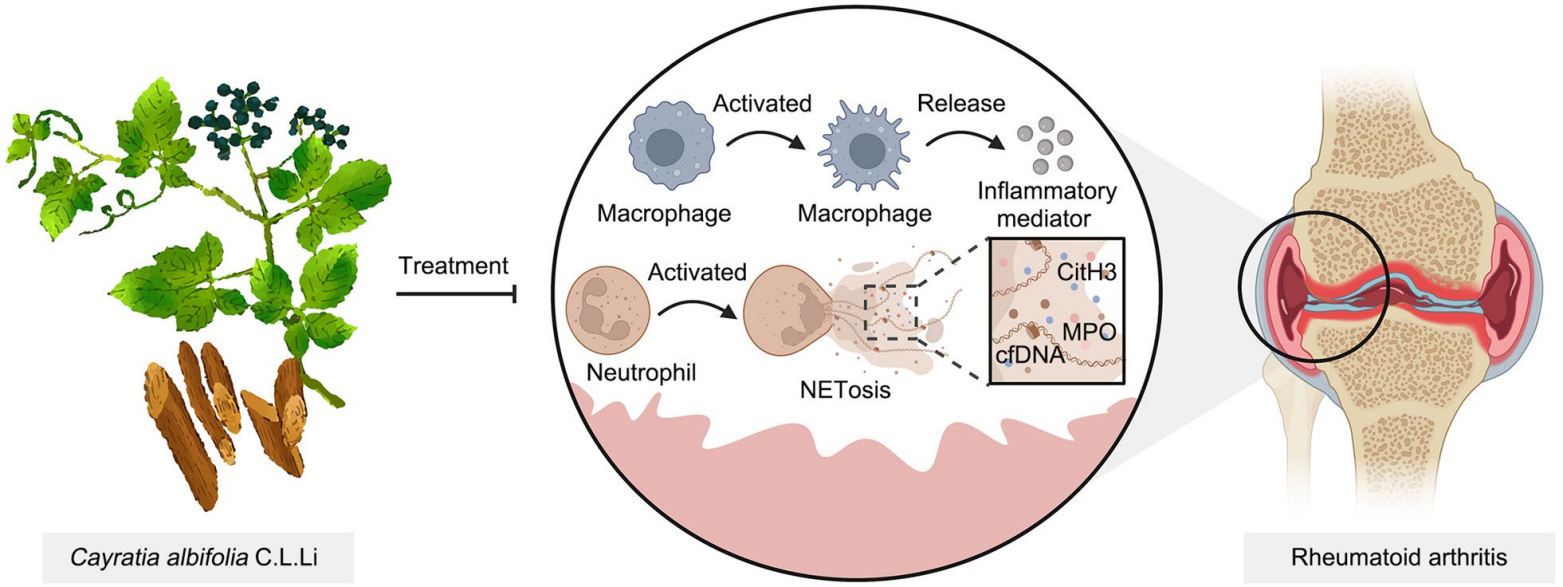

## Introduction

Rheumatoid arthritis (RA) is a chronic systemic auto-immune disease characterized by inflammatory cell infiltration to cause persistent joint destruction [1, 2]. RA has a prevalence of 1% in the world and the pathogenic mechanisms have not been definitely known until now [3, 4]. Synovial joint inflammation, bone destruction and autoimmune response are considered as major pathogenic risk factors of RA [5]. Although many agents are clinically used to treat RA, a great proportion of patients become refractory to current therapeutic regimens [6, 7]. Thus, the discovery of innovative drugs against RA is urgently needed.

Immune cell dysfunction is one of RA inducing factors [8]. In particular, previous study have shown that dysfunctions of macrophages and neutrophils are highly associated with RA progression. For example, pro-inflammatory macrophages (M1-type) contribute remarkably to the RA progression by secreting various types of inflammation mediators, such as interleukins [9]. Macrophages have also been implicated in the facilitation of bone resorption, resulting in perijoint osteoporosis and joint deformity [10]. Additionally, macrophages play a crucial role in modulating the function of T cells and B cells. Thus, it is an effective strategy to regulate the imbalance of the proportion of macrophages in RA treatment. CAC has been documented to attenuate zymosan A-induced inflammation by inhibiting the polarization of M1 macrophages [11]. Moreover, there have been reports highlighting the significant role of neutrophil extracellular traps (NETs) in the pathogenesis of RA [12–14]. NETs formation, a process termed NETosis, has been widely found in the synovium and peripheral blood of RA patients [13]. In particular, recent researches reveal that autoantibodies to citrullinated protein antigens (ACPAs) promote the early development of RA [13]. Meanwhile, citrullinated proteins were markedly produced in NETosis [15]. In mechanism, histones are catalyzed to citrullination that causes chromatin decondensation, thereby resulting in marked lattice DNA release to trigger acute cell death [16]. These cell-free DNA (cfDNA) was coated with nuclear proteins and cytosolic proteins, such as citrullinated histone H3 (CitH3) and myeloperoxidase (MPO), which accelerate RA progression [17–19]. Previous studies have documented that macrophage-derived inflammatory factors have a significant ability to induce NETosis in neutrophils, which is closely associated with RA [13]. Moreover, the release of NETs has the potential to enhance the production of inflammatory mediators in macrophages and initiate immune cell activation [13, 20, 21]. Thus, the discovery and development of effective agents specially regulating macrophages and neutrophils function is an attractive anti-RA direction in the future. Nowadays, traditional medicine derived from herbs has become a crucial complementary treatment for RA. Therefore, it is necessary to discover novel anti-RA agents with lower toxicity and high efficiency from traditional herbs. Vine plant *Cayratia albifolia* C.L.Li (CAC), also called Jiao-Mei-Gu (JMG), is a traditional herb to treat inflammatory diseases including rheumatic lumbago, acute sprain, and contusion in Dong people live of China [22]. Of note, modern studies have demonstrated that CAC extract significantly inhibited lipopolysaccharide (LPS)-induced inflammatory response in heart, liver, lung, kidney tissue by inhibiting NF-κB activation [22]. Moreover, chemical composition analysis indicated that JMG extract possesses several anti-inflammatory compounds, such as organic acids (palmitic acid), phenols (catachol), and terpenoids (betulinic acid) [23].

According to the Dong medicine theory, CAC can exert potential therapeutic effect on RA. However, no studies have explored the effect and underlying mechanisms of CAC in RA therapy. Herein, we will investigate the effect of CAC extract against RA to clarify the molecular targets and mechanisms in this study.

## Materials and methods

### Chemicals and reagents

High glucose Dulbecco’s Modified Eagle Medium (DMEM), trypsin and penicillin–streptomycin, were obtained from Macgene (Beijing, China). Fetal bovine serum (FBS) was from PAN-Biotech (Aidenbach, Germany). Complete/Incomplete Freund’s adjuvant and LPS were from Sigma-Aldrich (St Louis, MO, USA). Bovine type II collagen was from Macklin (Shanghai, China). Methotrexate was obtained from Bidepharm (Shanghai, China). 4% paraformaldehyde was from Bioroyee Biotechnology (Beijing, China). EDTA and DAPI were from Solarbio (Beijing, China). Goat anti-rabbit secondary antibody, endogenous peroxidases blocker and goat serum albumin were from ZSGB-Biotech ((Beijing, China). PMA are purchased from Med Chem Express (MCE). Hoechst 33342 was obtained from TargetMol (Shanghai, China). Sytox Green was from Beyotime (Shanghai, China). Primary antibody of IL-6 was from Bioss (Beijing, China). Primary antibody of TNF-α was from Proteintech (Chicago, IL, USA). Primary antibodies against PI3K/p-PI3K were from Bioworld (St. Louis Park, MN, USA). Primary antibodies against p-Akt and p-mTOR were bought from Cell Signaling Technology (Beverly, MA, USA). Primary antibody against Akt was from Biogot (Nanjing, China). Antibodies of mTOR, MPO, CitH3 were from Abcam (Cambridge, UK).

### Preparation of CAC extract

CAC plants were collected and authenticated by Hunan Provincial Key Laboratory of Dong Medicine in July 2021. The specimen was located at the herbarium of Modern Research Center for Traditional Chinese Medicine, Peking University (NO. CAC202106). The roots of CAC were soaked and boiled in 3 times volume of 95% ethanol for 1 h. Then, the boiled solution was filtered to extract filter residue once again. The filtrates were merged and concentrated by rotary evaporation. Finally, the concentrated extract was freeze-dried and stored at 4 °C.

### Identification of major chemical components of CAC extract

The CAC extract was analyzed by Exion LC AD liquid chromatography (SCIEX) coupled with a Triple TOF 6600 + mass spectrometer (SCIEX). The dissolved CAC extract was subjected to separation using a Waters Acquity HSS T3 Column (100 mm × 2.1 mm, 1.8 μm) followed by the introduction of the eluent into the mass spectrometer (MS). MS data acquisition was carried out under positive ion modes. The MS parameters were set as follows: pilot gas pressure at 55 psi, atomizing gas pressure at 55 psi, curtain gas pressure at 35 psi, ion source temperature at 600 °C, and positive ion spray voltage at 5500 V. The MS acquisition mode employed was information dependent. The MS acquisition range was as follows: primary MS, m/z 100–1500 Da; secondary MS, m/z 50–1500 Da. The data was analyzed by SIEVE software (Thermo Scientific) and compared with mzcloud and mzvault databases.

### Cell culture

Murine macrophages (RAW 264.7) were obtained from Peking Union Medical College Cell Bank (Beijing, China), and cultured in DMEM supplemented with 10% FBS, 100 U/ml penicillin and 100 μg/ml streptomycin at 37 °C with 5% CO_2_.

### Animals

Male Sprague–Dawley rats (6 weeks old, weight 180–220 g) were purchased from Vital River Laboratories (Beijing, China). The rats were housed in a room at 25 ± 1 °C under 12 h dark–light cycle. They were acclimated for 7 days and free access to food and water.

### Collagen-induced arthritis (CIA) model and drug administration

Following adaptive feeding, the rats' tail roots were subcutaneously injected with a 100 μl mixture containing Bovine type II collagen and complete Freund's adjuvant at a 1:1 ratio. After 7 days, the same tail roots of rats were injected subcutaneously with 100 μl mixture of Bovine type II collagen and incomplete Freund’s adjuvant at a 1:1 ratio to induce RA. After 7 days, CIA rats were assigned averagely into three groups, including CIA group (n = 6), CAC extract group (100 mg/kg) (n = 6), methotrexate group (MTX, positive group) (1 mg/kg) (n = 6). Rats without RA induction were control group (CON) (n = 6). The rats of CAC group were orally given 100 mg/kg CAC extract suspension in 0.5% sodium carboxymethylcellulose (CMC-Na) once a day. The MTX group was orally given 1 mg/kg MTX solution in 0.5% CMC-Na every 3 days. Isoflurane was used to anesthetize the rats, which were sacrificed on the 45th day of administration.

### Clinical evaluation of the severity of RA

The condition of joint erythema and swelling was scored every 7 days after 10 days of administration. The arthritis index (AI) score of one joint was ranged from 0 to 4: 0, no swelling and erythema; 1, slight joint swelling and mild erythema; 2, moderate swelling at ankle and erythema; 3, obvious swelling from ankle to toes and severe erythema; 4, severe swelling with inflammatory response in the whole foot limb.

### Measurement of hind paw volume

Throughout the drug treatment period, the volume of the rats' hind paw was measured at 7-day intervals, starting from 10 days of administration, using a toe volume measuring instrument (YLS-7C, Jinanyiyan Technology, Shangdong, China). Water was almost filled in the test tube on the instrument. After resetting the instrument reading, the hind limbs of rats were submerged into the test tube, resulting in an increase in the water level. Once the fluid level reaches the protrusion at the top of the ankle joint, while ensuring that the rat maintains a stationary position, the numerical value displayed on the instrument would undergo a change. Upon achieving a state of numerical stability, the instrument's display revealed the volume of the hind paw in the rats.

### Hematoxylin and eosin (H&E) staining

Following the sacrifice of the rats, the right ankle joints were isolated for histopathological examinations. The joints were fixed with a 4% paraformaldehyde solution and subsequently decalcified using a 10% EDTA solution. After the decalcification process, the ankle joints were embedded in paraffin and sliced into sections approximately 5 μm thick using a microtome. Then, the sections were stained by H&E with standard program. Finally, the sections were scanned using an WS-10 Digital Panoramic Scanner (Zhiyue, Jiangsu, China).

### Immunohistochemistry (IHC) analysis

The ankle joints were sliced to facilitate the subsequent procedures. Antigen retrieval of the sections was accomplished by subjecting them to microwave irradiation for a duration of 10 min. In order to suppress the activity of endogenous peroxidases, the sections were treated with H2O2 through the process of incubation. The samples were blocked by 5% goat serum albumin and incubated with primary rabbit anti-rat TNF-α/IL-6 antibody (1:200) overnight. After incubated with the goat anti-rabbit secondary antibody, the protein was detected used 3–3′-diaminobenzidine (DAB) substrate kit. The samples were stained with hematoxylin. Then, the sections were scanned using an WS-10 Digital Panoramic Scanner (Zhiyue, Jiangsu, China). The immunostaining level was quantified by Image-J.

### Preparation of CAC extract-crosslinked beads

FeCl_3_·6H_2_O (0.325 g), Na_3_Cit (0.20 g) and NaAc·3H_2_O (1.20 g) were dissolved in ethylene glycol and reacted at 200 °C for 8 h. Racemic-2–3-dimercaptosuccinic acid (15 mg) was added to the product, which was shaken for 1.2 h to synthesize sulfhydryl-bound Fe_3_O_4_ beads. Next, ethylester L-lysine triisocyanate (44.5 mg) and 4,4′-dihydroxybenzophenone (42.8 mg) were added to the product to synthesize 4,4′-dihydroxybenzophenone (DHBP)-bound Fe_3_O_4_ beads. Finally, CAC extract and DHBP-bound Fe_3_O_4_ beads were mixed and irradiated under UV (254–365 nm) at 25 °C for 1 h to produce CAC extract-crosslinked beads.

### Target protein identification with ‘Target-fishing’ strategy

As previously reported [24], CAC extract crosslinked-beads were added to LPS-treated RAW264.7 cell lysates, while 200 ng/ml CAC extract was added to one group of lysates for target protein competition. The mixture was incubated overnight at 4 °C. Proteins captured by CAC extract were eluted from beads, digested, and identified by liquid chromatography with a LTQ Velos pro mass spectrometer (Waltham, MA, USA).

### LC–MS/MS analysis of target proteins

The chromatographic separation of the samples was carried out using a C18 reversed-phase material column (75 μm × 10 cm, 3 μm particle size). Then, the eluent was flowed into the mass spectrometer (MS) with a flow rate of 300 nl/min. Mass spectrometric data of proteome was processed using Proteome Discover (1.4) software with the SEQUEST search engine (Thermo Scientific).

### Isolation of rat neutrophils

The Rat Peripheral Blood Neutrophil Isolation Solution Kit (Solarbio, Beijing, China) was utilized for the isolation of rat neutrophils from peripheral blood. Rat peripheral blood was collected in the heparin anticoagulant tube. Following manufacturer’s protocol, neutrophils were isolated by centrifugation at 1000 rcf for 20 min at 25 °C. Erythrocytes mixed with neutrophils were lysed with red blood cell lysis in the kit. Then the purified neutrophils were washed with PBS.

### Wright-Giemsa stain assay

Neutrophils were stained with Wright-Giemsa Stain Kit (Jiancheng Bio-Engineering Institute, Nanjing, Jiangsu, China). Air-dried neutrophilic granulocytogram was incubated with Wright-Giemsa solution R1 for 1 min. Then, Wright-Giemsa solution R2 was added to mix with R1 by shaking slides. After incubating for 5–8 min, the solution was sucked out and the smear was washed with flowing water for 30 s. The images of cell staining were visualized by a light microscope (IX73, Olympus, Japan).

### Quantification of cfDNA

Purified neutrophils were treated with PMA (1 μM) for 4 h with CAC extract (50, 100, 200 μg/ml) or not. The culture solution was centrifugated to collect supernatant. The content of cfDNA in supernatant was detected using dsDNA HS Assay Kit (Yeasen, Shanghai, China). The supernatant was incubated with dsDNA Reagent for 2 min at 25 °C. Finally, the value of fluorescence intensity of the samples was measured using a fluorescence spectrophotometer (PerkinElmer, Waltham, MA, USA) (Ex = 480, Em = 520). The dsDNA HS Assay Kit (Yeasen, Shanghai, China) contains Standard A1 and Standard A2, which respectively serve as the cfDNA control reagent and standard reagent. The fluorescence intensity value A1 of Standard 1 is indicative of a concentration of 0 ng/μl of cfDNA, while the fluorescence intensity value A2 of Standard 2 corresponds to a concentration of 10 ng/μl of cfDNA. The cfDNA concentrations in each sample were calculated using the equation: Concentration _cfDNA_ = (10-A1) A/A2 + A1, where A represents the fluorescence intensity values for each sample group.

### Immunofluorescence assay of cfDNA

Neutrophils were treated with PMA (1 μM) for 4 h with CAC extract (50, 100, 200 μg/ml) or not. Then, Sytox Green (1 μM) and Hoechst 33342 (1 μM) were added to the supernatant and incubated for 20 min. Fluorescence was visualized (488 nm/519 nm for Sytox Green; 340 nm/488 nm for Hoechst 33342) by a Zeiss microscope (LSM880, Oberkochen, Germany).

### Immunofluorescence assay of MPO and CitH3

The bottom of the confocal dishes was coated with Poly-D-lysine overnight. After washing with PBS, the confocal dishes were dried at 37 °C. Purified neutrophils were seeded onto the dishes and cultured at 37 °C. After 30 min of neutrophils adhered to the bottom of confocal dishes, they were treated with PMA (1 μM) for 4 h with CAC extract (50, 100, 200 μg/ml) or not. After incubated with primary rabbit anti-rat MPO/CitH3 antibody (1:200/1:1000) overnight, the neutrophils were incubated with the Alexa Fluor 488-labeled secondary antibody for 1 h and DAPI (10 μg/ml) for 20 min. Fluorescence was visualized (488 nm/519 nm for GFP; 340 nm/488 nm for DAPI) by a Zeiss microscope (LSM880, Oberkochen, Germany).

### Nitric oxide (NO) assay

The quantification of NO content was accomplished with NO Assay Kit (Jiancheng Bio-Engineering Institute, Nanjing, Jiangsu, China). RAW264.7 cells were incubated with LPS (1 μg/ml) for 24 h with CAC extract (50, 100, 200 μg/ml) or not. Then, cell supernatant collected was incubated with Griess reagent I and II for 10 min. Then, supernatant of mixture collected by centrifugation was incubated with Griess reagents III, IV and V for 10 min. Finally, the absorbance value of mixture was detected by a microplate reader (Tecan, Ausserfeld, Switzerland) at a wavelength of 570 nm.

### Real-time PCR analysis

RAW264.7 cells were treated with LPS (1 μg/ml) for 6 h with CAC extract (50, 100, 200 μg/ml) or not. MolPure Cell/Tissue Total RNA Kit (Yeasen, Shanghai, China) was used to extract the RNA. Then, cDNA was obtained from reverse transcription using Hifair III 1st Strand cDNA Synthesis SuperMix. The cDNA was amplified with Hieff qPCR SYBR Green Master Mix. The result of real-time PCR would provide CT value. The relative mRNA level of certain genes was 2^−ΔΔCT^ by normalizing to GAPDH. The primers were listed in Table 1.

**Table 1:** Primers for real-time PCR

Gene	Sequence
IL-6	F: 5′-CACAGAAGGAGTGGCTAA-3′
	R: 5′-CCATAACGCACTAGGTTT-3′
iNOS	F: 5′-CCGAAGCAAACATCACATTCA-3′
	R: 5′-GGTCTAAAGGCTCCGGGCT-3′
MCP-1	F: 5′-CTTCTGGGCCTGCTGTTCACAGTT-3′
	R: 5′-TTCTTGGGGTCAGCACAGACCTCT-3′
GAPDH	F: 5′-GGTGAAGGTCGGTGTGAACG-3′
	R: 5′-CTCGCTCCTGGAAGATGGTG-3′

### Western blot analysis

The RAW 264.7 cells were lysed using RIPA containing 1% protease inhibitor for 30 min. Proteins were separated by 6%-10% SDS-PAGE gels and transferred to PVDF membranes. The membranes were blocked for 30 min in 5% (w/v) skimmed milk solution at 25 °C. Then, the membranes were incubated with the primary antibodies overnight at 4 °C and the secondary antibodies for 1 h at 25 °C. Protein blots were developed using enhanced chemiluminescence (ECL) and visualized using Tanon 5200 Imaging Analysis System (Tanon, Shanghai, China).

### Statistical analysis

All experiments were performed at least three times. Statistical significance was completed with one-way analysis of variance (ANOVA) by using GraphPad Prism 8.0 software. All results were expressed as mean ± standard error of mean (SEM). A value of *P* < 0.05 was considered statistically significant.

## Results

### The main chemical components of CAC extract

**Fig. 1:**
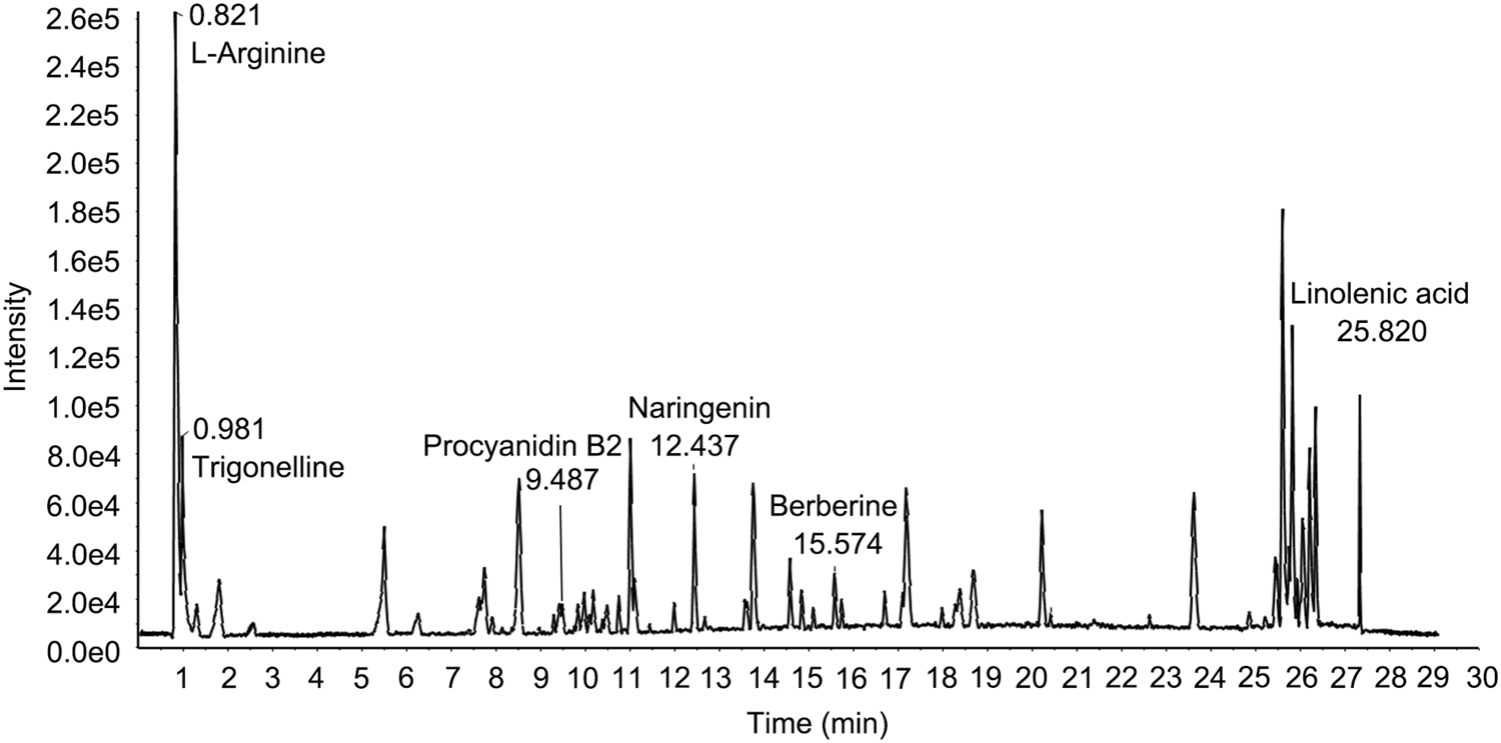
The main chemical components of CAC extract

To elucidate the composition of the main chemical components in the CAC extract, we conducted an analysis using liquid chromatography-tandem mass spectrometry (LC-MS/MS). The analysis of the CAC extract revealed the presence of six compounds with significant content, as indicated by the base peak chromatogram (BPC) (Fig. 1). The identified compounds in the CAC extract were as follows: 1. L-Arginine, 2. Trigonelline, 3. Procyanidin B2, 4. Naringenin, 5. Berberine, 6. Linolenic acid.

### CAC extract prevented arthritis progression in CIA rats

**Fig. 2:**
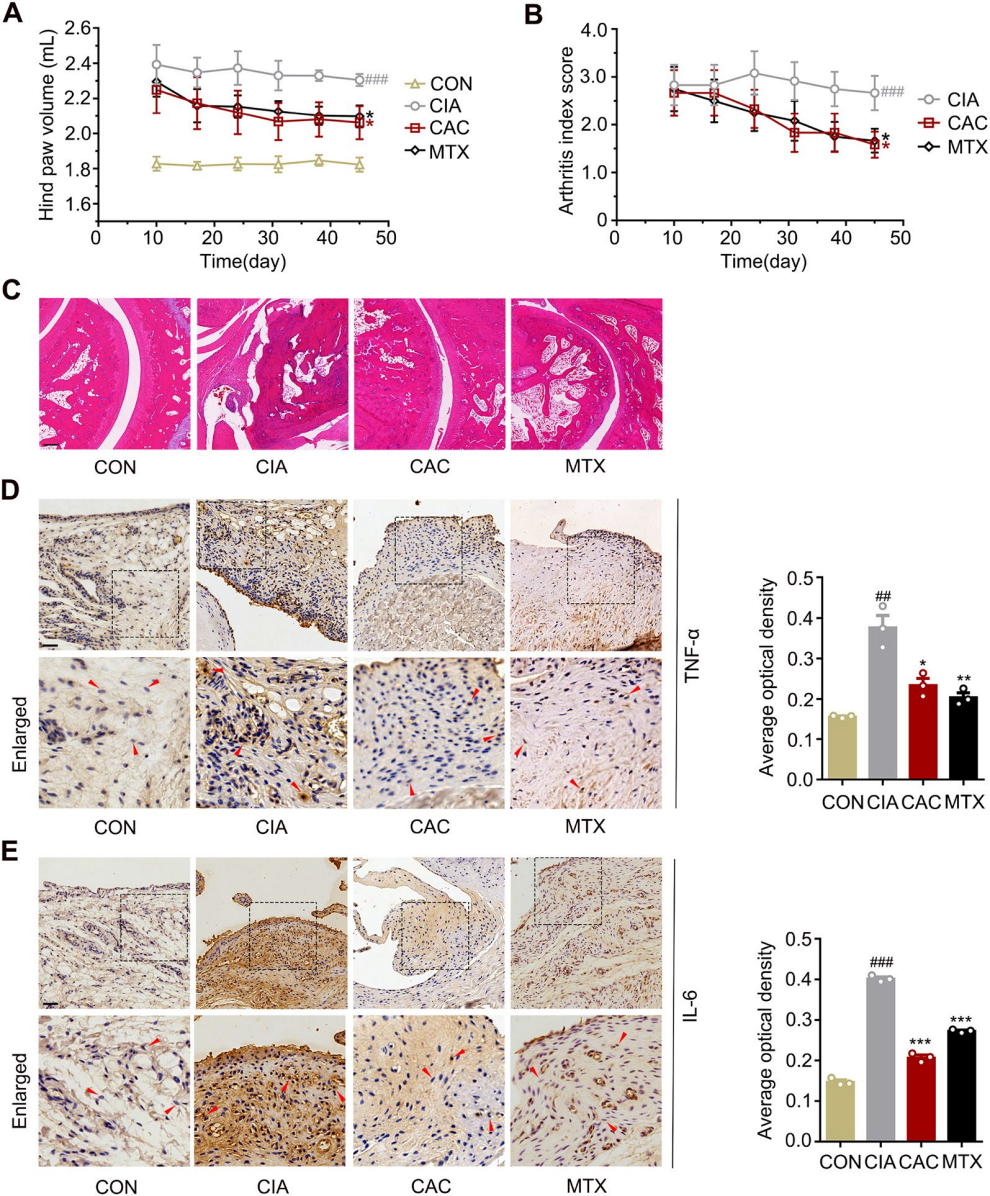
CAC extract prevented arthritis progression in CIA rats. **A, B** The hind paw volume (**A**) and arthritis index (AI) score (**B**) of hind paws in rats from: CON group (n = 6), CIA group (n = 6), CAC group (100 mg/kg/day, n = 6), MTX group (1 mg/kg/3 days, n = 6). **C** H&E staining of each group (scale bars: 50 μm). **D–E** IHC analysis of the expression of TNF-α (**D**) and IL-6 (**E**) in each group (scale bars: 25 μm). ^*^P < 0.05 *vs*. CIA group; ^**^P < 0.01 *vs*. CIA group; ^***^P < 0.001 *vs*. CIA group; ^##^ *P* < 0.01 *vs*. control group; ^###^ *P* < 0.001 *vs*. control group

To assess the potential anti-RA properties of CAC extract, a rat model of collagen-induced arthritis (CIA) was established. The CIA model exhibits a plethora of pathological and immunological features that closely resemble those observed in human RA [25]. It is widely recognized as the accepted benchmark for in vivo investigations pertaining to RA [26]. Rats were immunized with the mixture twice, with a 7-day interval between each immunization. Following the second immunization, rats exhibited distinct arthritic symptoms, such as foot redness, swelling, and double-stepped joints, within 7 days. Compared with the CIA group, CAC extract effectively alleviated RA severity. 45 days after administration, the hind paw volume of CIA rats was larger than that of normal control rats (*P* < 0.001), but the hind paw volume of the rats treated with CAC was remarkably reduced compared with CIA rats (*P* < 0.05) (Fig. 2A). Moreover, the arthritis index (AI) score of CIA rats was higher than that of normal control rats (*P* < 0.001), but CAC treatment significantly decreased the AI score compared with CIA rats (*P* < 0.05) (Fig. 2B). The rats with treatment of methotrexate (MTX) showed decrease in the hind paw volume and AI score similar to CAC group. H&E staining showed severe architecture destruction of joint in CIA rats, with articular cartilage damage, synovial hyperplasia and inflammatory cells infiltration. However, CAC extract showed significantly inhibitory effects on destruction of joint architecture similar to MTX (Fig. 2C). The results of immunohistochemical (IHC) staining showed that TNF-α and IL-6 expression in the joint synovial tissue of CIA rats were obviously upregulated compared with normal control rats. However, the TNF-α and IL-6 expression in rats with treatment of CAC extract or MTX were effectively decreased compared with CIA rats (Fig. 2D, E). Collectively, these findings suggested that CAC extract treatment alleviated RA in rats and inhibited inflammatory response.

### Identification of molecular targets of CAC extract via ‘Target-fishing’ strategy

**Fig. 3:**
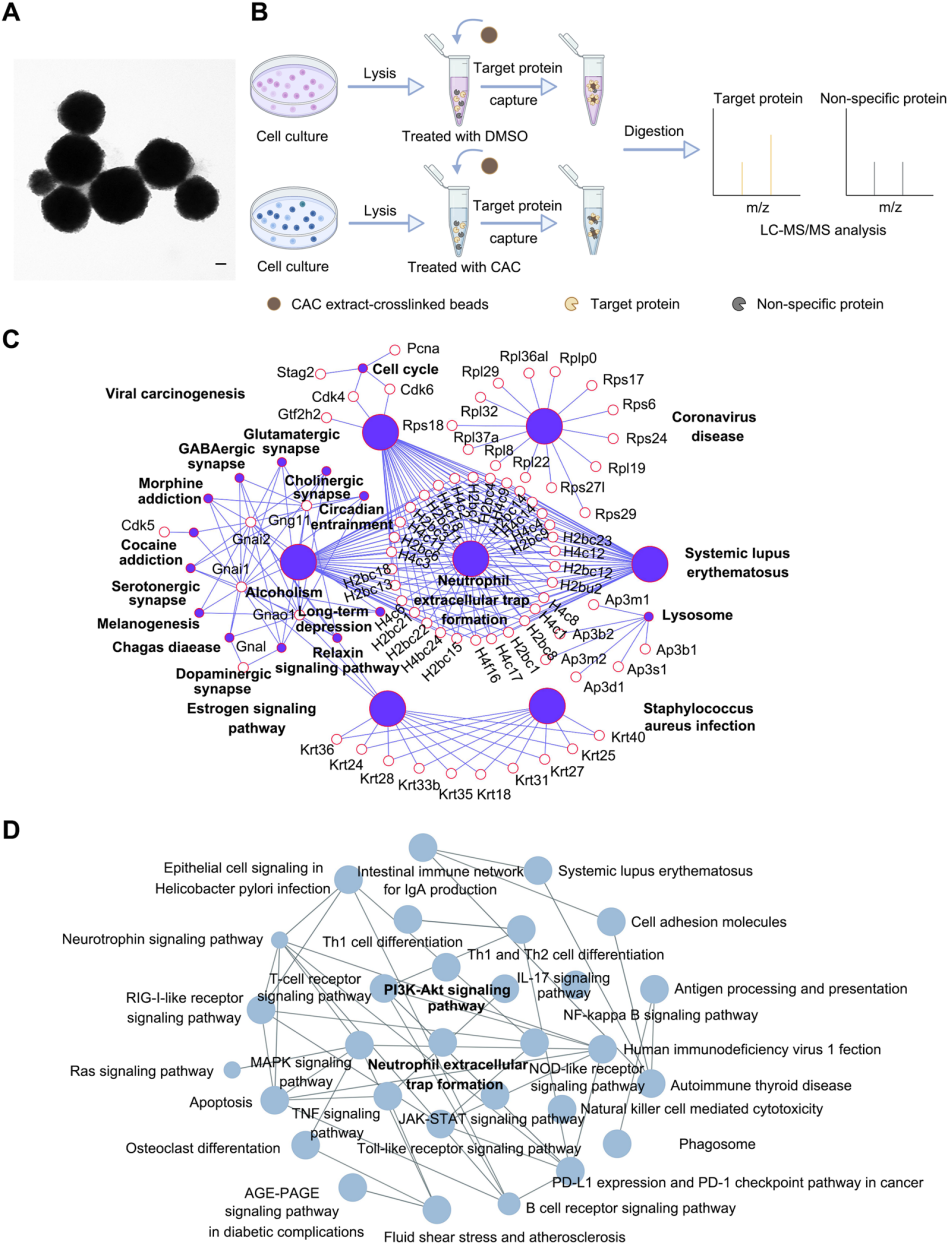
Identification of pharmacological targets of CAC extract via ‘Target-fishing’ strategy. **A** Representative transmission election microscopy (TEM) image of Fe_3_O_4_ beads (scale bar, 500 nm). **B** Workflow of target identification of CAC extract based on pull-down/MS. **C, D** The pathway-gene network analysis of 177 potential target proteins (**C**) and proteins associated with RA (**D**) on Cytoscape

To investigate the pharmacological mechanism of CAC extract against RA, we attempted to capture molecular target proteins of CAC extract using ‘Target-fishing’ strategy in macrophages. We synthesized CAC extract-crosslinked beads via UV-induced crosslinking reaction (ZY strategy) as described previously (Fig. 3A) [24]. The crosslinked beads containing CAC-extract were incubated with cell lysates from LPS-activated RAW 264.7 cells, with or without the presence of CAC extract, to assess target binding competition. The binding proteins were eluted from the beads, followed by trypsin digestion of the protein samples. Subsequently, the digested samples were analyzed using LC–MS/MS (Fig. 3B). The enrichment analysis on signaling pathways of target proteins was performed by Cytoscape. Based on the LC–MS/MS results, we applied a filtering criterion where proteins with a binding versus competition ratio greater than 1 were retained, leading to a final set of 177 proteins. Subsequently, the names of these proteins were entered into the ClueGo plugin within the Cytoscape software for conducting KEGG pathway analysis (Fig. 3C). Then, the genes associated with different terms in the KEGG results are ranked based on their relevance. Significantly, the NETs signaling pathway exhibited a prominent ranking and demonstrated a close association with the pathogenesis and treatment of RA. Consequently, our hypothesis proposes that the formation of NETs constitutes the primary signaling pathway of utmost importance. As shown in Fig. 3C, neutrophil extracellular trap (NETs) formation was the most key signaling pathway. Furthermore, 423 genes involved in RA were picked out on OMIM and Genecards database with relevance score greater than 10. The signaling pathway analysis also showed that NETs formation played a crucial role (Fig. 3D). Thus, we speculated that CAC may alleviate RA progress via inhibiting the formation of NETs.

### CAC extract inhibited neutrophils extracellular traps (NETs) formation

**Fig. 4:**
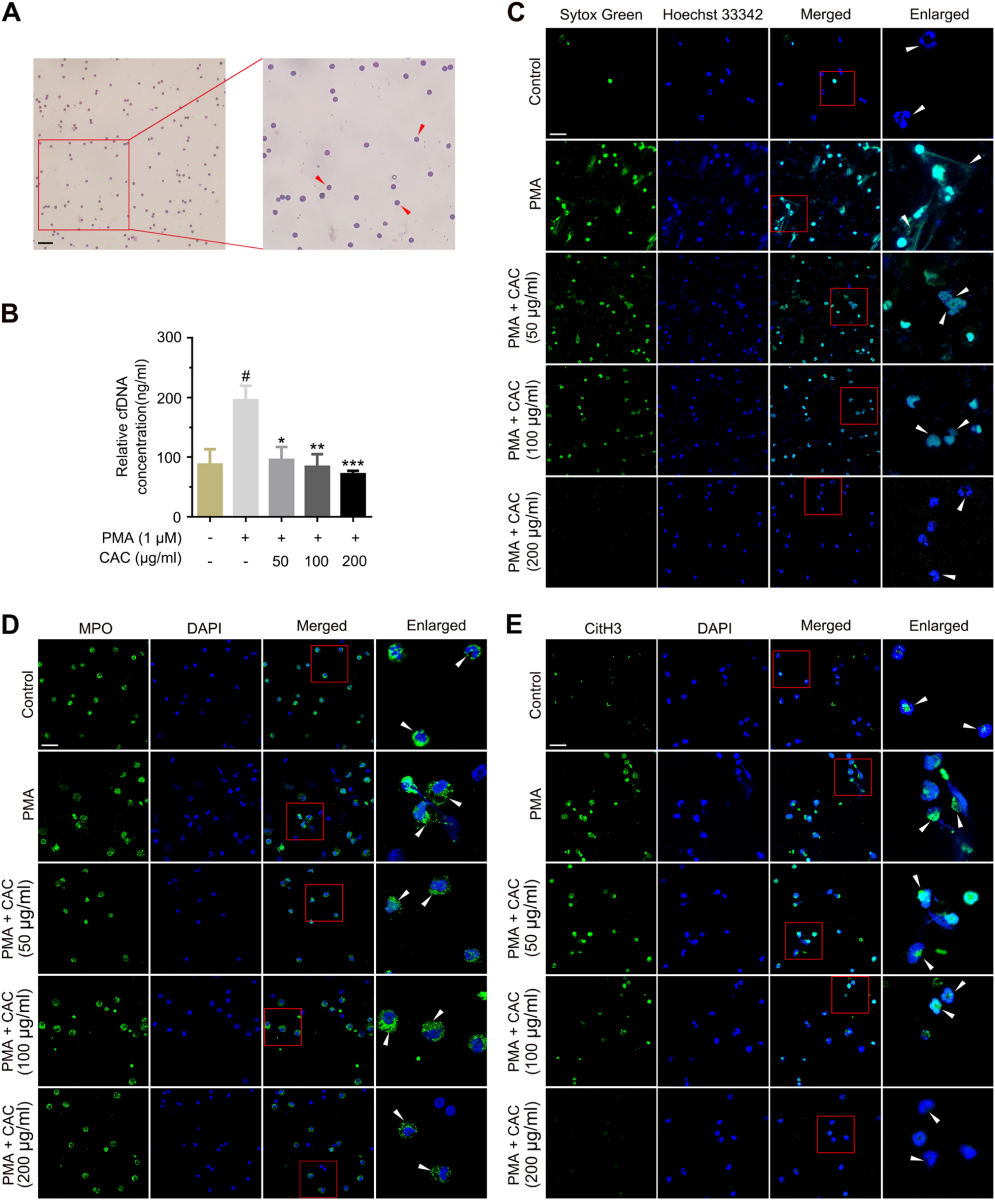
CAC extract inhibited neutrophils extracellular traps (NETs) formation. **A** Representative image of purified neutrophils after Wright-Giemsa’s staining (scale bar, 100 μm). **B** CAC extract inhibited PMA-induced cfDNA release in neutrophils. **C–E** Representative immunofluorescence image of cfDNA (**C**), MPO (**D**) and CitH3 (**E**) staining of neutrophils (scale bar, 100 μm). PMA-induced (1 μM) cfDNA release in neutrophils was inhibited by CAC extract (50, 100, 200 μg/ml). The expressions of MPO and CitH3 induced by PMA (1 μM) in neutrophils were suppressed by CAC extract (50, 100, 200 μg/ml). *P < 0.05 *vs*. PMA group; **P < 0.01 *vs*. PMA group; ***P < 0.001 *vs*. PMA group; ^#^*P* < 0.05 *vs*. control group

In order to provide additional verification regarding the impact of CAC extract on the functionality of the previously identified targets, we proceeded to purify neutrophils from the peripheral blood of rats. Wright-Giemsa stain verified the purity of neutrophils (greater than 95% pure) (Fig. 4A). Neutrophils exhibit a spherical morphology, and upon staining, the granules present in their cytoplasm manifest a light lavender-red coloration. NETs are released by neutrophils to capture and kill bacteria and other pathogens. The process of its formation involves histone citrullination, chromatin decondensation and extracellular release of decondensed chromatin, which containing cfDNA, MPO and CitH3 and so on. In in vitro experiments, NETs is often motivated using PMA. To examine the effect of CAC extract on NETosis, we measured PMA-induced cfDNA release in neutrophils with CAC extract or not. The CAC extract effectively suppressed the noticeable elevation in PMA-induced cfDNA release (Fig. 4B). To further evaluate the effect of CAC extract on NETosis, we quantitatively visualized the effect of CAC extract on PMA-induced cfDNA release and protein markers expression in neutrophils by immunofluorescence assays. As shown in Fig. 4C, nuclei stained by Sytox Green and Hoechst 33342 showed being decondensed in neutrophils treated with PMA compared with normal control neutrophils, which was significantly restrained by CAC extract. CAC extract also significantly inhibited the release of MPO and CitH3 induced by PMA, while MPO and CitH3 were marked in green and the nuclei were marked in blue with DAPI (Fig. 4D, E). These findings confirmed that CAC extract remarkably inhibited the formation of NETs. CAC prevented RA progression by inhibiting the formation of NETs.

### CAC extract inhibited macrophage-mediated inflammation response via PI3K-Akt pathway

**Fig. 5:**
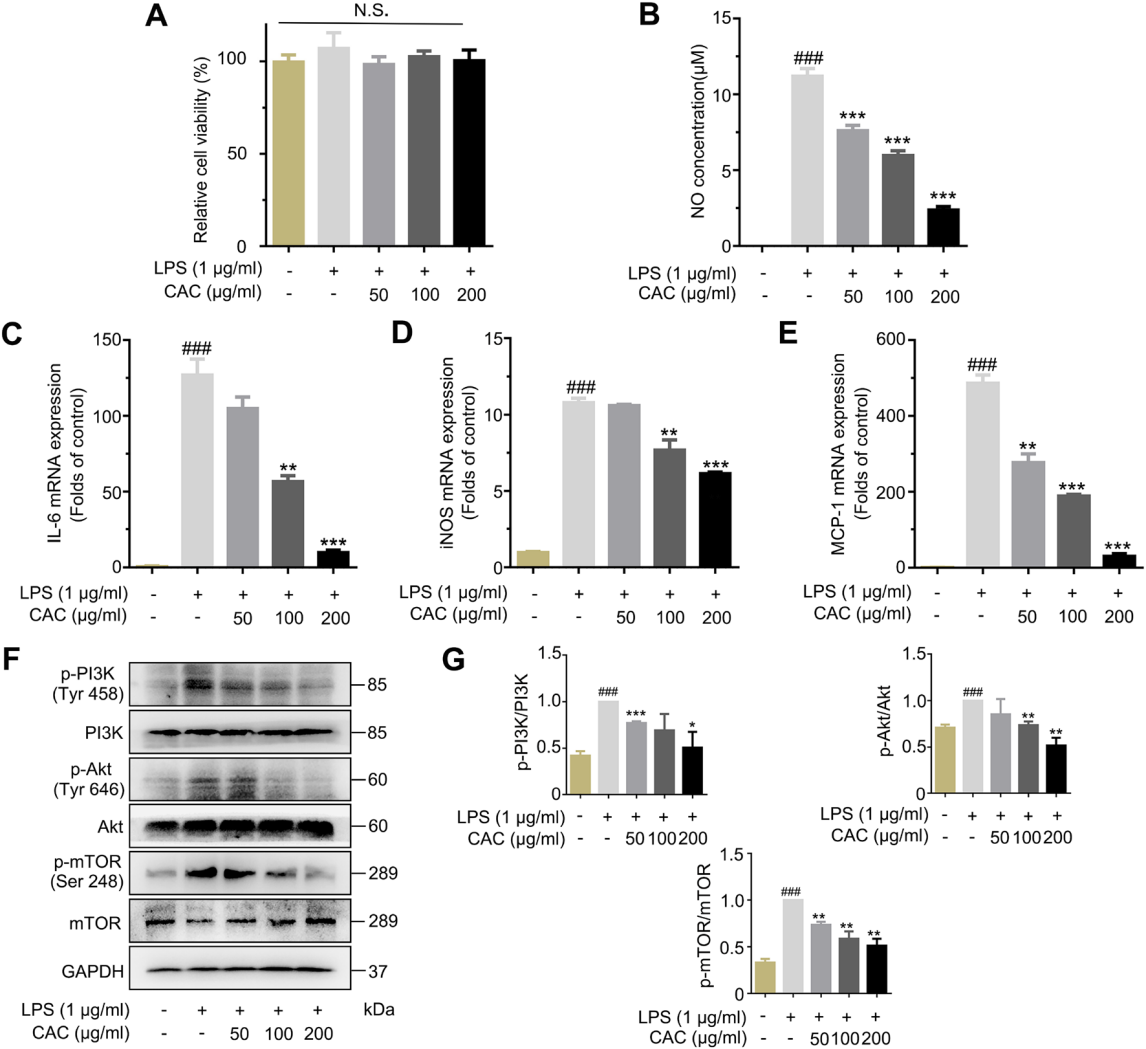
CAC extract inhibited macrophage-mediated inflammation response via PI3K-Akt pathway. **A** LPS (1 μg/ml) with or without CAC (50, 100 and 200 μg/ml) did not affect cell viability. **B** CAC extract attenuated NO production. **C–E** CAC extract decreased mRNA expression levels of IL-6 (**B**), iNOS (**C**) and MCP-1 (**D**). **F** CAC extract inhibited PI3K-Akt-mTOR pathway in LPS-activated RAW264.7 cells. **G** Quantitative analysis for relative phosphorylation levels of PI3K (p-PI3K), Akt (p-Akt) and mTOR (p-mTOR) were performed by normalizing to model group. *P < 0.01 *vs*. LPS group; **P < 0.01 *vs*. LPS group; ***P < 0.001 *vs*. LPS group; ^##^ *P* < 0.001 *vs*. control group; ^###^ *P* < 0.001 *vs*. control group; N.S., not significant by ANOVA with Dunnett’s *post-hoc* test

In order to assess the anti-inflammatory properties of the CAC extract in macrophages, we conducted an analysis to examine its impact on the production of inflammatory mediators in RAW 264.7 cells stimulated with LPS. The preliminary trial suggested that the optimal effectiveness of CAC extract was observed at concentrations of 50, 100, and 200 µg/ml, and it showed a notable dose-dependent relationship. MTT assay was also performed to assess the viability of RAW264.7 cells upon stimulation with CAC extract. Our findings demonstrated that the exposure of RAW264.7 cells to concentrations of 50, 100, and 200 μg/ml of CAC extract did not elicit significant effects on cell viability (Fig. 5A). Moreover, the CAC extract significantly attenuated the noticeable increase in NO release induced by LPS (Fig. 5B). IL-6, iNOS and MCP-1 are crucial inflammatory factors released by macrophage in the development of inflammation. Thus, we investigated the effects of CAC extract on mRNA expression of IL-6, iNOS, and MCP-1. As shown in Fig. 5C, D and E, the mRNA expressions of IL-6, iNOS and MCP-1 induced by LPS were effectively suppressed by CAC extract. According to KEGG analysis, the PI3K-Akt signaling pathway has a pivotal role in RA. Additionally, this pathway plays a crucial role in guiding macrophage responses to inflammatory stimuli by controlling macrophage polarization [27, 28]. The PI3K-Akt pathway also regulates macrophage survival, migration, and proliferation [27]. Further literature review on *Cayratia japonica,* a plant within the same genus as CAC, suggests its potential in suppressing inflammatory responses through modulating the PI3K/Akt signaling pathway [23]. We then examined the effect of CAC extract on LPS-activated PI3K-Akt-mTOR pathway phosphorylation. Western blot data showed that CAC extract markedly blocked the phosphorylation states of PI3K (p-PI3K), Akt (p-Akt) and mTOR (p-mTOR) (Fig. 5F, G). These data suggested that CAC extract could effectively inhibit the inflammatory response in macrophage via PI3K-Akt pathway in a concentration-dependent manner.

## Discussion

RA is an autoimmune disorder that is currently incurable [29, 30]. The existing treatment options for RA include non-steroidal anti-inflammatory drugs (NSAIDs), glucocorticoids (GCs), and disease-modifying anti-rheumatic drugs (DMARDs). However, these treatments have limited efficacy and often lead to severe side effects [31, 32]. The application of clinical therapy for RA raises concerns regarding the increased risk of infections and financial burden [33, 34]. Therefore, there is an urgent need to discover new and effective drugs for the treatment of RA. Traditional Chinese medicine (TCM) is recognized as a substantial resource for the treatment of RA owing to its multi-component and multi-target characteristics [35, 36]. TCM offers distinct advantages such as low incidence of side effects and cost-effectiveness in disease management [37]. Consequently, the identification of effective TCM options for RA therapy deserves considerable attention.

As a traditional medicine of Dong minority in China, CAC has been used to treat inflammatory disorders for a long time [11]. Within the realm of Dong medicine, CAC exhibits a notable therapeutic effect on RA [22]. However, the efficacy and underlying mechanisms of CAC in RA therapy have yet to be demonstrated and explored, consequently restricting its widespread application. To ascertain the impact of CAC, a CIA rat model was constructed. Moreover, previous investigations utilizing CAC water extract at approximately 100 mg/kg have demonstrated a significant inhibitory effect on inflammation response induced by LPS and paw edema caused by zymosan A in mice, without any notable adverse effects [11, 22]. Therefore, in this experiment, we employed a similar concentration range to explore the effectiveness and underlying mechanisms of CAC alcohol extract in the treatment of RA. The result indicated that the CAC extract exhibited a significant reduction in hind paw volume and also alleviated inflammatory responses. Our findings indicate that the effectiveness of CAC extract in treating RA is comparable to that of the positive control, MTX. Notably, CAC extract offers the advantage of circumventing the potential adverse effects associated with MTX treatment, including hepatotoxicity, infections, and gastrointestinal complications [38]. Studies have shown that a significant proportion (30–40%) of patients with RA do not achieve optimal results and often develop tolerance to MTX treatment [39, 40]. Consequently, the diverse chemical compounds found in CAC extract may have the potential to offer improved therapeutic effects, akin to those observed with combined medication approaches.

Subsequently, in order to capture the proteome-wide target proteins of CAC extract from macrophage cell lysates, we utilized CAC extract-crosslinked beads. This technique exploits the carbon radicals on the surface of beads, activated by UV light, to form covalent bonds with organic molecules from plant extracts. It is particularly well-suited for identifying the cellular targets associated with multi-molecule systems, such as traditional Chinese herbal medicine. Synthetic beads were confirmed to undergo successful cross-linking with chemical extracts through Fourier transform infrared analysis. Furthermore, electron microscopy evaluation revealed that the beads were uniformly dispersed with a regular spherical shape. The target identification capability of the beads was also validated through surface plasmon resonance technology, demonstrating a high binding affinity between compounds and target proteins [24]. Thus, this method demonstrates robust stability and reliability in target identification. The result of KEGG analysis revealed that the molecular mechanisms of CAC extract in the treatment of RA may be involved in the formation of NETs. The excessive autoimmune response triggered by NETosis is a critical pathogenic mechanism underlying RA. Consequently, the inhibitory effect of CAC extract on NETosis may serve as a crucial molecular mechanism for its anti-RA properties. Subsequently, we conducted immunofluorescence staining of cfDNA, MPO, and CitH3 to validate the notable inhibition of NETosis upon treatment with CAC extract. Previous studies have documented significant neutrophil infiltration in the synovial membranes of arthritis-afflicted mice, with neutrophil depletion effectively impeding the progression of RA [41–43]. Consequently, targeting dysregulated neutrophils emerges as a crucial therapeutic approach for managing RA. It is worth mentioning that various natural small molecules have been documented for their ability to alleviate RA by inhibiting NETosis [12, 44, 45]. However, the therapeutic potential of these compounds is marred by considerable toxicities, such as nephrotoxicity, hepatotoxicity, and reproductive toxicity [12, 46]. In contrast, the CAC extract offers a unique advantage as it combines multiple compounds, resulting in a broader spectrum of biological effects with minimal toxicity. This favorable characteristic positions CAC extract as a promising candidate for broader applications in RA therapy. Nevertheless, in order to facilitate broader clinical application, further investigations are required to identify the bioactive compounds present in CAC. The implementation of standardized quality control measures is of paramount importance. The resolution of these concerns requires ongoing exploration.

In conclusion, our study provides confirmation of the therapeutic effects and underlying pharmacological mechanism of CAC extract in mitigating the inflammatory response associated with RA. Our findings provided convincing evidence that targeting neutrophils can be an effective strategy in RA treatment, and CAC can effectively inhibit excessive NETosis induced by neutrophils activation to treat RA. Meanwhile, the elucidation of molecular mechanism provides the basis for CAC extract in RA clinical treatment.

## Data Availability

Data and material will be made available on request.
